# Supplementary material for: Structural Model of the Bilitranslocase Transmembrane Domain Supported by NMR and FRET Data
Source: PLoS One. 2015 Aug 20;10(8):e0135455. doi: 10.1371/journal.pone.0135455 (PMC4546402; doi:10.1371/journal.pone.0135455)
Supplement: S1 File — Binding parameters for transmembrane TM2 and TM3 segments in SDS micelle by FRET experiments (Table A). Hydrodynamic parameters by DOSY spectroscopy (Table B). 15N relaxation rates for 15N-labeled alanines in TM2, TM3 peptides and TM2:TM3 pairs extracted from NMR data (Table C). CD spectra (Fig A). Distance between Pro85 and Pro231 in TM2 and TM3 fragments of BTL protein (Fig B). 1H-15N HSQC spectra (Fig C). FT PGSE analysis of DPFGDSTE experiment for SDS micelle, TM2, TM3, and TM2:TM3 mixture. (Fig D). Spatial structure of studied species from NMR data. (Fig E). Experimental R 1 and R 2 relaxation rates for 15N-labeled alanines in TM2 fragment (Fig F). Experimental R 1 and R 2 relaxation rates for 15N-labeled alanines (Ala225 and Ala233) in TM3 (Fig G). Experimental R 1 and R 2 relaxation rates for 15N-labeled alanines (Ala225' and Ala233') in minor conformation of the TM3 (Fig H). Experimental values of 15N R1, R2 relaxation rates and {1H}-15N NOE (Fig I). Results of analysis of 15N relaxation data for TM2 and TM3 fragments (Fig J). (DOCX) [file pone.0135455.s001.docx]

**Structural model of the Bilitranslocase transmembrane domain supported by NMR and FRET data**

Amrita Roy Choudhury^1^, Emilia Sikorska^2^, Johannes van den Boom^3^, Peter Bayer^3^, Łukasz Popenda^4^, Kosma Szutkowski^4,5^, Stefan Jurga^4,5^, Massimiliano Bonomi^6,7^, Andrej Sali^7^, Igor Zhukov^4,8*^, Sabina Passamonti^9*^, and Marjana Novič^1*^

^1^National Institute of Chemistry, Hajdrihova 19, 1001 Ljubljana, Slovenia

^2^Faculty of Chemistry, University of Gdańsk, Gdańsk, Poland

^3^Institute for Structural and Medicinal Biochemistry, Center for Medical Biotechnology, University of Duisburg-Essen, Essen, Germany

^4^NanoBioMedical Center, Adam Mickiewicz University, Poznań, Poland

^5^Faculty of Physics, Adam Mickiewicz University, Poznań, Poland

^6^Department of Chemistry, University of Cambridge, Cambridge, United Kingdom

^7^Department of Bioengineering and Therapeutic Sciences, Department of Pharmaceutical Chemistry, and California Institute for Quantitative Biosciences, University of California, San Francisco, California, USA

^8^Institute of Biochemistry and Biophysics, Polish Academy of Sciences, Warsaw, Poland

^9^Department of Life Sciences, University of Trieste, Trieste, Italy

*** Corresponding authors**

**E-mail:** [**marjana.novic@ki.si**](mailto:marjana.novic@ki.si)

[**spassamonti@units.it**](mailto:spassamonti@units.it)

[**igor@ibb.waw.pl**](mailto:igor@ibb.waw.pl)

**Keywords:** transmembrane peptides, bilitranslocase, molecular dynamic simulations, NMR spectroscopy

**Abstract**

We present a 3D model of the four transmembrane (TM) helical regions of bilitranslocase (BTL), a structurally uncharacterized protein that transports organic anions across the cell membrane. The model was computed by considering helix-helix interactions as primary constraints, using Monte Carlo simulations. The interactions between the TM2 and TM3 segments have been confirmed by Förster resonance energy transfer (FRET) spectroscopy and nuclear magnetic resonance (NMR) spectroscopy, increasing our confidence in the model. Several insights into the BTL transport mechanism were obtained by analyzing the model. For example, the observed *cis-trans* Leu-Pro peptide bond isomerization in the TM3 fragment may indicate a key conformational change during anion transport by BTL. Our structural model of BTL may facilitate further studies, including drug discovery.

**Introduction**

Understanding the molecular mechanisms underlying the passage of ions and small molecules through biological membranes is a fundamental aspect of cell physiology. This knowledge is also crucial for analyzing disease associations, for identifying potential drug targets, and for improving safety and efficacy of new or existing drugs. Transmembrane proteins provide key means of molecular transport through the cell membrane. They are also extensively studied as potential drug targets. Of strong interest as potential drug targets are the organic anion transporter family proteins (OATPs), due to their capacity to serve as tumor biomarkers and effective cancer drug transporters [1,2]. The physiological expression patterns of the OATPs are altered in malignant tissues. Screening tumors for OATP expression may enable an OATP-targeted therapy with higher efficacy and, most importantly, decreased side effects relative to current therapies. In addition to offering new opportunities, the membrane transport proteins also pose challenges to drug discovery research. To overcome the challenges and difficulty of determining membrane protein structures, several methodologies for studying membrane proteins have been reported, including specialized techniques for stabilizing and manipulating proteins, which depend on the protein itself and the method planned – X-ray crystallography, NMR spectroscopy or other techniques to explore protein structure [3].

Motivated by an interest in druggability of anion transporters, we have identified the transmembrane protein bilitranslocase [4] (BTL) (UniProt O88750, TCDB 2.A.65) as a potential drug target that exhibits partial functional similarity to OATPs [5]. BTL is a plasma membrane transporter involved in the transport of organic anions, including the transport of bilirubin through the liver plasma membrane [6]. BTL may play an important role in both human pathology [7] and drug delivery [8]. The primary structure and biological functions of BTL have been known and studied for decades [4-14]. However, the secondary and tertiary structures of the BTL are not known. No sequence homologs have been detected for BTL, however, it has been predicted, based on homology-independent considerations, that BTL has four TM α-helices [15]. In addition, NMR spectroscopy has validated the α-helical structures of two key transmembrane helices, TM2 and TM3, in the SDS media [16,17]. H-bonds were suggested to play a role in the transport mechanism, based on chemometrics modeling studies of substrates with experimentally determined affinities [8,11]. Despite these characterizations, a full understanding of the BTL transport mechanism is hindered by the lack of its complete atomic structure(s). Although BTL may exists in a multimeric form [10], the complete oligomeric state of the protein is not clearly known, thus making it further difficult to understand the complete structural assembly of BTL.

The structure of a membrane protein can be characterized by a variety of approaches. If good quality crystals can be obtained, X-ray crystallography provides an accurate atomic structure [18]. NMR spectroscopy can be used to map the flexible segments and their conformational dynamics [19], including the *cis*–*trans* heterogeneity [20]. However, the 3D structure determination of membrane proteins by X-ray crystallography or NMR spectroscopy remains challenging, for a number of reasons [18]. Besides taking the advantage of the emerging experimental technologies targeted towards membrane protein structural biology [21], computational analysis, with extrapolation of sparse experimental information, is a valuable complementary approach in expanding our knowledge of transmembrane protein structures [22]. It includes sequence dependent predictions of transmembrane regions, their stability and interactions, as well as molecular dynamics simulations, coarse-grain simulations and other stochastic methods.

Here, we aimed to structurally characterize the transmembrane domains of BTL. Coarse-grained models of the assembly of the four transmembrane α-helices, TM1, TM2, TM3, and TM4, of BTL were generated using Monte Carlo (MC) simulations, a stochastic simulation process useful for systems with several coupled degrees of freedom, considering predicted transmembrane helix-helix interactions and several other restraints. The generated conformations were clustered and ranked based on the Discrete Optimized Protein Energy (DOPE) statistical potential for inter-atomic distances in proteins [23]. The top scoring models were analyzed to propose the packing of the four BTL transmembrane helices by structural modeling relying on experimental constraints.

To validate the proposed model, we used nuclear magnetic resonance (NMR) and Förster resonance energy transfer (FRET) spectroscopies to map interactions between TM2 and TM3, the two transmembrane segments that participate in the transport of anions through BTL [9,16,17]. Structural analysis of the TM2:TM3 pair in the SDS-d_25_ micelles was performed based on the previously collected NMR data [16,17]. The existence of the TM2:TM3 pair in the SDS micelles was demonstrated by the FRET experiments. To increase the quality of our experimental data sets, we introduced two ^15^N-labeled alanines (^15^N-Ala) into the TM2 and TM3 segments. The selective ^15^N-Ala labeling allowed us to observe the dynamics of TM2, TM3, and the TM2:TM3 pair in the SDS micelles. The occurrence of a *cis-trans* peptide bond isomerization was detected in the TM3 fragment at the Leu230–Pro231 peptide bond. This isomerization may be key to the uptake mechanism of different anions *via* the BTL.

**Results and Discussion**

**Transmembrane helix-helix interactions**

The four TM α-helices (TM1: Phe24-Asp48, TM2: Phe75-Cys94, TM3: Gly220-Tyr238, TM4: Pro254-Ser276) have been predicted by a neural network model based on homology-independent considerations [15]. The final transmembrane region boundaries were predicted based on statistically derived amino acid preference data for the transmembrane region boundary positions. Although charged residues are present at the boundaries, our previous studies have shown that these boundary residues are contained within the lipid bilayer during the MD simulations, and do not show any translational motion along y-axis [16,17]. These results were confirmed by NMR spectroscopy [16,17]. The transmembrane helix-helix interactions were predicted taking into consideration the correct topology of BTL. This prediction is independent of the previously determined structures of TM2 and TM3.

The four transmembrane helices of BTL can in principle form six combinations of helix-helix pairs. Each transmembrane helix was represented as a rigid body. Models that optimize pairwise helix-helix interactions were enumerated with the open-source *Integrative Modeling Platform* (IMP) package (<http://integrativemodeling.org>) [24]. All configurations of all combinations of rotational, translational and tilting degrees of freedom, within specified ranges and at specified resolution that were guided by previous statistical analyses of transmembrane protein structures were considered. The relative stability of each helix-helix configuration was estimated using a scoring function based on known interaction data on transmembrane helices. The BTL transmembrane helix pairs TM2-TM3 and TM1-TM4 that showed the most optimized configurations, as denoted by their lowest scores, were reported to be interacting.

The TMhit algorithm predicts helix-helix interactions based on residue contacts with at least one pair of predicted residue contacts supporting each helix-helix interaction [25]. Considering only the residue contacts predicted with more than 75% certainty, TMhit also predicted the TM2-TM3 and TM1-TM4 transmembrane helix-helix pairs to be interacting. For the TM2-TM3 pair, the predicted residue contacts are Thr81-Gln223 (100% certainty), Thr81-Cys224 (96%), Pro85-Val222 (82%), and Cys76-Ala225 (81%). The only contact predicted with more than 75% certainty for the TM1-TM4 pair is His43-Pro258 (79%).

Incidentally, the two interacting transmembrane regions TM2 and TM3 contain the AxxxG and GxxxxxxA sequences, respectively. The GxxxG and GxxxxxxG motifs are known transmembrane helix dimerization motifs where Gly can be replaced by other small amino acid residues, such as Ala or Ser [26]; thus, they may include the sequences in TM2 and TM3. The motifs are further associated with β-branched residues Val90, Ile228, Ile232, and Ile234. The β-branched amino acid residues isoleucine and valine are hypothesized to reduce the entropic cost of transmembrane protein folding with their constrained rotameric freedom in the helical conformation [27]. The presence of these transmembrane helix dimerization motifs in association with the β-branched amino acid residues in TM2 and TM3 provides additional validation for the predicted TM2-TM3 transmembrane helix-helix interaction pair.

**Predicted Arrangements of the Four Transmembrane Regions of BTL.**

Monte Carlo simulations were used to sample the accessible conformational space and predict the probable assembly of the four transmembrane regions of BTL. The diameter of the assembly was restrained to ~26 Å. The tilt and depth (translation along z-axis) restraints were calculated based on the length of the transmembrane helices. Other restraints applied include DOPE, excluded volume, packing, and distance restraints corresponding to the predicted TM2-TM3 and TM1-TM4 transmembrane helix-helix interactions. The restraints are based on previous analyses of known transmembrane protein structures [28]. Besides the rotational and tilting movements, each individual transmembrane helix was allowed only certain translations. Accordingly, for TM1 the center of mass was fixed and the only allowed translation was along the z-axis. For TM2, both y- and z-axis translations were allowed. For TM3 and TM4, translations along all three axes were allowed.

Two million conformations of the four BTL transmembrane regions were generated by the Monte Carlo method. These conformations were then clustered by hierarchical clustering based on their pairwise Calpha RMSD differences. The threshold of 2 Å on the RMSD cutoff resulted in 3520 clusters; the centroids of these clusters were selected as the representative conformations.

The BBQ algorithm was used to reconstruct the atomistic backbones of the cluster representations [29]. The side chains were added using the SCWRL4 algorithm [30]. The representative all-atom structures were then ranked by their DOPE scores developed for transmembrane proteins (G.-Q. Dong and M. Bonomi, unpublished) and further analyzed to infer how the four transmembrane regions of BTL are arranged.

There are six possible unique arrangements of the four transmembrane regions of BTL as observed from the extracellular surface (Fig. 1A). The transmembrane regions TM1, TM2, TM3, and TM4 are denoted as *A*, *B*, *C*, and *D*, respectively. Positioning TM1 at the top-left corner and reading clockwise, the six arrangement types are *ABCD*, *ADBC*, *ACDB*, *ABDC*, *ACBD*, and *ADCB*. The 3520 representative conformations from the Monte Carlo simulation were classified into these six groups (Table 1). The most frequently observed arrangement is *ABDC* (Top2, 3, 4 conformations in Fig. 1B). Arrangement types *ACBD* and *ADCB* (Top5 and Top1 conformations, respectively, in Fig. 1B) are also frequented. The arrangements of the amino acid residues around the helical axes (Fig. 1C) support the predicted packaging of the transmembrane helices.

------ Fig. 1 --------

We scored the 3520 all-atom representations using the DOPE statistical potential for transmembrane proteins. The distribution of the 100 top-scoring conformations among the arrangement types is similar to that of all 3520 conformations (Table 1). Forty-four out of the 100 top-scoring conformations are *ABDC*. This arrangement has the interacting transmembrane helices positioned diagonally opposite to each other (Top2, 3, 4 in Fig. 1B). The highest scoring conformation exhibits the *ADCB* arrangement (Fig. 1B). The fifth ranked conformation is *ACBD*. Both the *ADCB* and *ACBD* arrangements have the interacting helices positioned adjacent to each other. These three-arrangement types together account for 86 of the 100 highest-scoring conformations and 84.4% of all 3520 conformations. In conclusion, our analysis helped to narrow down the probable arrangements of the four transmembrane regions of monomeric BTL. However, it has not resulted in a precise model of the functional transport channel. Keeping in mind that there are some experimental evidences about a possibility of dimeric or trimeric form of BTL [10], further experimental investigations will be needed.

------ Table 1 --------

**Distance between the Transmembrane Regions TM2 and TM3.**

The TM2 transmembrane region lies immediately adjacent to the conserved bilirubin-binding motif. TM3, on other hand, is adjacent to a second bilirubin-binding site and also contains a conserved ligand-binding motif at the C-terminal. Therefore, the transmembrane regions TM2 and TM3 are postulated to play key roles in the formation of the transport channel, ligand binding, and mediation.^9,12^ Previous experiments with cysteine and arginine modifications had concluded that BTL exists in two metastable forms with different substrate affinities [10]. This metastable nature of BTL, detected experimentally [10], can possibly be accounted to the presence of proline-induced kinks, which may render flexibility to both transmembrane regions. The Pro85 in TM2 and Pro231 in TM3 are located in the middle of the transmembrane channel and presumably define the pore in variable functional states. Therefore, we analyzed the distances between the proline residues and the N-termini of TM2 and TM3, as follows.

The most populated arrangement *ABDC* has the transmembrane regions TM2 and TM3 arranged diagonally opposite to each other. The average distance between Pro85 in TM2 and Pro231 in TM3 is 19 ± 4.3 Å for the 44 top-scoring *ABDC* conformations, and 17.4 ± 4.35 Å for all 1330 *ABDC* conformations (Figs. 2A, 2C). The proline-proline distance in 36% of the conformations with the *ABDC* arrangement is between 16 and 20 Å. In *ADCB* and *ACBD*, the average proline-proline distance is 14 ± 3.6 Å and 13.1 ± 3.4 Å, respectively. In these two arrangements, the TM2 and TM3 transmembrane regions are adjacent to each other. The distance between the N-termini of the two transmembrane segments is 30.3 ± 4.1 Å for all three arrangement types (Figs. 2B, 2D). In *ABDC*, this distance represents the diagonal in the complete assembly of the four transmembrane regions.

------ Fig. 2 --------

**Identification of TM2:TM3 Pair in SDS Micelles with FRET Technique.**

The integration of TM2:TM3 pair in the SDS detergent was confirmed by the Förster Resonance Energy Transfer (FRET) experiment. The energy transfer from an excited state of a donor fluorophore to an acceptor fluorophore should occur typically when they are in the proximity (<10nm). Atto488 and Atto594 were used as FRET dye donor-acceptor pair to label the TM2 and TM3 peptide fragments. Based on the long-range dipole-dipole coupling mechanism, the FRET signal was recorded for the surfactant (SDS) with dye-labeled TM2 or TM3 peptides. This signal was much stronger than that recorded for the dye-free sample, thus establishing the proximity between the two fragments TM2 and TM3 in a single SDS micelle (Fig. 3). Moreover, the collected data demonstrate the presence of both types of pairs (TM2:TM2 and TM3:TM3) in the surfactant micelles characterized with the dissociation constants *K*_d_ = 346.1, 71.85, and 190.6 μM for TM2:TM3, TM2:TM2, and TM3:TM3, respectively (Table A in S1 File). It is interesting to note that the TM2:TM3 dimer had the lowest *K*_d_, thus lowest interaction strength. As a consequence the TM2:TM3 dimer might enable cis-trans isomerization and individual flexibility, which was also shown by Monte Carlo simulations. The Förster radius for the Atto488–Atto594 pair is estimated as 60 Å, which is comparable to the determined hydrodynamic radius (*R*_h_) [31] of the SDS-d_25_ micelle. Thus our measurements are not suitable either for establishing the relative positions of TM2 and TM3 fragments inside the SDS micelle or for estimating the distances between fragments in the surfactant environment. However, they undoubtedly confirm the existence of TM2:TM3 pairs in the micelle, which indicates the interactions between the TM2 and TM3 fragments.

------ Fig. 3 --------

**CD Spectroscopy of TM2:TM3 Pair in SDS Micelle Media.**

The CD spectra recorded for isolated TM2 and TM3 fragments and for the TM2:TM3 pair in SDS and DPC surfactants exhibit a substantial amount of α-helical conformation characterized by two minima near 208 and 222 nm (Figure A (left) in S1 File) [32]. A quantitative analysis suggests that in zwitterionic (DPC) environment the fraction of time and/or residues of TM2 and TM3 in the α-helical conformation is 15% and 40%, respectively (Figure A (left) in S1 File).

Inspection of CD data reveals that, similar to previously reported data for megainin peptides [33], preference of α-helical conformation of TM2 and TM3 segments are different in anionic (SDS) and zwitterionic (DPC) media, reflecting the importance of electrostatic and hydrophobic interactions between the peptides and micelles. For the TM3 fragment, the helical conformation is favored in the anionic media, whereas the TM2 segment prefers such form in the zwitterionic micelle. The conformational behavior obtained for TM2:TM3 pair is determined by the TM2 fragment rather than the TM3 one in all type of used media (Figure A (right) in S1 File).

**Structural Analysis of BTL TM2:TM3 Pair in SDS-d_25_ Micelle by NMR Spectroscopy.**

Structural details of the two key BTL transmembrane regions, TM2 and TM3, in SDS-d_25_ micelle were recently analyzed on the basis of homonuclear and heteronuclear 2D NMR data sets [16,17]. The high-resolution 3D structures were solved based on 250 (139 intraresidual, 78 sequential, and 33 medium range) and 180 (107 intraresidual, 58 sequential, and 15 medium range) nontrivial ^1^H–^1^H NOE distance constraints obtained from 2D NOESY spectra analysis of TM2 and TM3, respectively. 3D structures as obtained from our previous studies [16,17] were positioned in the SDS lipid media and subjected to MD simulations in water bath with AMBER 9 molecular dynamics package (Fig. 4) [34].

To detect the intermolecular NOESY contacts between the TM2 and TM3 transmembrane fragments, which are critical for structural analysis, two ^15^N-labeled alanines were incorporated in each of the studied peptides. The alanines Ala80 and Ala88 in TM2 and Ala225 and Ala233 in the TM3 fragment were labeled. Unfortunately, the 3D ^15^N-edited NOESY-HSQC spectra acquired with mixing times up to 250 ms did not exhibit any intermolecular signals due to the fact that TM2 and TM3 segments are separated by at least 13 Å (Figure B in S1 File), which is in line with the results obtained from the computational analysis of transmembrane region assembly. Finally, the 3D structure of the TM2:TM3 dimer in SDS micelles was evaluated based on previously determined experimental constraints for TM2 [17] and TM3 [16] fragments (Fig. 4).

--------------------- Fig. 4 ------------------------

An overlay of 2D ^1^H-^15^N HSQC spectra acquired for TM2 and TM3 fragments shows that the chemical shifts ^1^H and ^15^N of amide groups for ^15^N-labeled alanines are in agreement with previously recorded data sets acquired on natural abundance of the ^15^N isotope [16,17]. Furthermore, the ^1^H-^15^N HSQC spectra of the TM2:TM3 pair are presented as a superposition of those obtained with individual TM2 and TM3 segments (Fig. 5, Figure C (left, right) in S1 File). Additional peaks corresponding to a less populated conformation were detected for the TM3 species (Fig. 5A). Inspection of 2D ^1^H-^1^H NOESY spectra acquired for the TM2:TM3 pair reveals a weak signal, which could be assigned as Leu230 ^1^H^α^-Pro231 ^1^H^α^ of the *cis* rotamer of the Leu230-Pro231 bond in the TM3 peptide (Figure C (bottom) in S1 File). We conclude that the second conformation appears due to the *cis-trans* isomerization of the peptide bond around the central Pro231 in TM3 fragment. The relative fraction of the second conformation was estimated to be 30% based on the ratio of peaks. Interestingly, this ratio did not change during the entire time of NMR observation, which suggests the presence of a high energy barrier between the two different conformers. Indeed, BTL is found to be present in two meta-stable conformers with different substrate affinities and the conversion between the conformers can be accelerated by presence BTL substrates, including bilirubin [10]. On the other hand, presence of a minor *cis* conformation was not detected for the central Ser84–Pro85 peptide bond in the TM2 segment.

------ Fig. 5 --------

The position of the TM2:TM3 pair in SDS micelles was analyzed based on the 25 ns trajectory simulated in AMBER 9 with the *parm99* force field and the TAV protocol (S1 File). As follows from the solved 3D structure of TM2:TM3 pair, the TM3 peptide takes a more central position inside the micelle, positioning itself in a more hydrophobic environment in comparison with previously published data [16]. At the same time, the TM2 fragment moves close to the micelle surface with the two α-helical parts buried in the hydrophobic region of the SDS micelle and the central loop around Pro85 is situated in the hydrophilic surface (Fig. 4). The translational diffusion coefficient (*D_tr_*) was determined with a DPFGDSTE (Double Pulsed Field Gradient Double Stimulated Echo) experiment. On the basis of this experimentally determined *D_tr_*, we estimated the hydrodynamic radius (*R*_h_) for all studied systems (Figure D, E, and Table B in S1 File). Since the bilirubin-binding motif (65-75) is close to (and part of) the transmembrane segment, interrupted by Pro85, it could be possible that binding of bilirubin at the extracellular surface of the protein triggers a conformational change of TM2 around this Pro kink. In fact, bilirubin and nicotinic acid are positive allosteric effectors, inducing the BTL into its high-affinity state [10]; in turn, this could be a driving factor for substrate transport. Flexibility around this Pro kink could also be triggered by cysteine reagents, which are certainly exposed to the medium (unpublished data) and induce BTL to its low-affinity conformation [10].

**Molecular Mobility of the TM2 and TM3 Fragments in SDS Micelles.**

The incorporation of two ^15^N-labeled alanines in the TM2 and TM3 fragments enabled the exploration of the dynamics of the peptide backbone in a residue specific manner by measuring three relaxation parameters that characterize the backbone ^15^N nuclei. Longitudinal (*R*_1_) and transverse (*R*_2_) relaxation rates were extracted with high accuracy from experimental data (Figures F-H in S1 File). The obtained *R*_1_ and *R*_2_ values together with steady-state {^1^H}-^15^N *NOEs* (Figure H in S1 File) allowed us to apply the isotropic rotation model in the *model-free* approach.^35^

Experimental magnitude of *R*_1_ relaxation rates falls in region 1.3 – 1.5 (s^-1^) for all studied species (Figure I in S1 File). Estimated values of *R*_2_ relaxation rates are comparable in case of three out of four alanines examined in TM2 and TM3 segments. The Ala225 in the TM3 fragment clearly shows a decreased relaxation rate *R*_2_, which dropped to 5 s^-1^ when compared with 8.2 s^-1^ detected for Ala233 (Table C and Figure I in S1 File). Finally, the {^1^H}-^15^N *NOE*s, evaluated with relatively lower accuracy, reveal moderately higher values for TM2 (0.65 – 0.55) than for TM2 peptide (0.6 – 0.45) (Table C and Figure I in S1 File). The relaxation data, acquired for the TM2:TM3 pair in SDS micelle, are similar to those obtained for the separate TM2 and TM3 fragments. The measured {^1^H}-^15^N *NOE* values for Ala80 and Ala233 in the TM2:TM3 pair could suggest more restrictive motions on the *ps*–*ns* time scale compared to the individual components.

The parameters of backbone dynamics (*S*^2^, τ_m_) were calculated for ^15^N-labeled alanines with *model-free* approach taking into account an isotropic diffusion model. The values of overall correlation times (τ_R_) were extracted from *R*_1_/*R*_2_ ratios (Figure J in S1 File); the values of 4.99 ± 0.05, 6.97 ± 0.08, and 6.87 ± 0.03 *ns* were obtained for TM2, TM3, and TM2:TM3 peptides, respectively. The *model-free* analysis clearly demonstrated more stable structure for TM2 peptide. The *S*^2^ values obtained for TM2 individual fragment in SDS surfactant were 0.98 and 0.86 for Ala80 and Ala88, respectively. In case of TM2:TM3 assembly the *S*^2^ decreased to 0.84 and 0.70 for Ala80 and Ala88, respectively, but still remained in the range characteristic for folded proteins (Figure J in S1 File). The TM3 segment exhibited lower values of *S*^2^ parameter, namely 0.51 and 0.78 for Ala225 and Ala233, respectively. In the TM2:TM3 pair similar values of *S*^2^ for both ^15^N-labeled alanines (Figure J in S1 File) were obtained. The relaxation time analysis demonstrated that the dynamic processes in TM3 fragment were substantially different from those in TM2. Intensive high frequency motions in *ns* – *ps* range were observed in TM3 peptide ascribed to Ala225 at the N-terminal part, which were detected neither in TM2 nor in the C-terminal part of TM3 in SDS micelle.

**Concluding Remarks**

In this work, we computationally analyzed the assembly of the four α-helical transmembrane regions of BTL, restricted by the predicted transmembrane helix-helix interactions TM2-TM3 and TM1-TM4. Of the six possible ways in which the transmembrane regions could be arranged, we have identified the three most probable arrangement types using Monte Carlo simulations. The most observed arrangement has the key transmembrane segments, TM2 and TM3, positioned diagonally opposite to each other. The distances between these two transmembrane regions were analyzed and are supported by NMR spectroscopy results.

Furthermore, the structure of the TM2:TM3 pair is analyzed in more detail using several experimental methods. The existence of the TM2:TM3 pair in an ionic SDS micellar environment and interaction between them was supported by results from NMR spectroscopy and FRET efficiency. Additionally, the 40 ns molecular dynamics simulations indicated the existence and stability of the TM2:TM3 pair in SDS micelle.

The kinks observed in the two key BTL transmembrane segments at the positions of the two central prolines (Pro85 in TM2 and Pro231 in TM3) could be one of the important structural aspects explaining the mechanism of transport of different anions by BTL. We observed two TM3 conformers in SDS solution, differing in the *cis* and *trans* rotamer of the Leu230–Pro231 peptide bond. Therefore, we can conclude that the proline kink in TM3 renders the flexibility to the transmembrane region. The transition between these two states could facilitate the molecular uptake process. This hypothesis is further supported by the strong conformational exchange processes at the N-terminal part of TM3, appearing probably due to the rotation around the Leu230–Pro231 peptide bond. This rotation could lead to the changes within the TM2:TM3 pair or even in the whole four-helix bundle, in turn leading to the transition between the two distinct functional states, as observed in the native membrane environment [10].

A comparison of the 3D structures of TM2:TM3 pair in SDS micelles with previously solved structures of individual fragments demonstrates remarkable changes in the position of peptides in the micelle. TM3 in the TM2:TM3 pair is likely positioned more deeply in the hydrophobic part of the SDS micelle than in a sample of TM3 alone [16]. The detailed structure of an individual α-helix likely depends on the neighboring helices; additional experimental data is needed to confirm the structure of this four-helix bundle.

To understand the arrangement of the BTL transmembrane helices and the structure of the transport channel at atomic level, it is essential to have knowledge of the exact oligomeric state of the protein, which is still lacking. Here, we made key steps towards achieving this goal. We suggested the probable assembly of the transmembrane regions of BTL. We also characterized the structure of the two functionally important transmembrane regions, TM2 and TM3, which in turn led us to hypothesize about conformational changes involved in the transport of ligands through the channel.

**Materials and Methods**

**Synthetic TM2 and TM3 Peptides.**

Synthetic peptides TM2B and TM3 (Ser73 – Leu99 and Gly220 – Tyr238, respectively), corresponding to the two key transmembrane segments of BTL, were purchased from CASLO Laboratory, Denmark (www.caslo.com) and were subjected to NMR and FRET experiments. Both peptides were synthesized as lyophilized trifluoroacetate salts. To avoid problems during synthesis, purification and NMR sample preparation, the extreme hydrophobicity of the peptides was moderated by adding four lysine residues (a LysTag KKKK) at the C-termini. The amino acid sequences of synthetic TM2B and TM3 are ^73^SSFCLFVATLQSPFSAGVSGLCKAILL^99^KKKK and ^220^GSVQCAGLISLPIAIEFT^238^KKKK, respectively, with purity higher than 93.8%. To measure the relaxation parameters with heteronuclear NMR spectroscopy, the alanines of both TM2B and TM3 fragments were ^15^N-labeled (^15^N-Ala80, ^15^N-Ala88 ^15^N-Ala225, and ^15^N-Ala233).

**Predicting Transmembrane Helix-Helix Interactions.**

The interactions between transmembrane helix-helix pairs in bilitranslocase were predicted using the open-source IMP program (<http://integrativemodeling.org>) [24] as well as the TMhit web server [25].

The IMP predictions take into account the complete transmembrane regions and not the individual residues. In this case, the BTL transmembrane region sequences with defined topologies served as the input. The rigid body representation of each transmembrane region was generated considering DOPE [23], excluded volume and packing restraints. The DOPE statistical potential for transmembrane proteins was developed and used internally at the SaliLab. These rigid body representations do not take into consideration the structures determined by either NMR or MD methods in our previous studies. Because transmembrane helices can interact in multiple ways [36], all possible transmembrane helix-helix pairs of BTL were considered and their conformations were optimized. Only the best scoring transmembrane pairs were regarded as interacting and considered for further analysis.

The TMhit algorithm [25] was used to predict the transmembrane helix-helix interactions based on residue contacts. It incorporates contact propensities, physiochemical and structural information to predict contact residues and their pairing relationships or helix-helix interactions. Previously predicted transmembrane regions and their topologies served as the input for the algorithm.

**Monte Carlo Sampling.**

The probable arrangements of the four BTL transmembrane regions were explored using MC sampling. The initial configurations of the four BTL transmembrane regions, considering only the CA atoms, were constructed based on the conformations generated from previous MD simulations [16,17]. The transmembrane region sequences, topologies and loop connectivity served as the input parameters. The primary restraints were defined by the predicted transmembrane helix-helix interactions and the filter on the crossing angles [37]. Added restrictions were applied on the transport channel diameter, the tilt and depth (translation along z-axis) of the transmembrane helices [28,36]. This discrete conformation space, defined by the applied restrains, was then sampled using the Monte Carlo method with varying temperatures.

**NMR Spectroscopy and 3D Structure Evaluation.**

The translational movements of the TM2 and TM3 transmembrane fragments and TM2:TM3 pairs in SDS-d_25_ micelles were characterized using DOSY technique with 64 gradients utilized on an Agilent VNMRS 600 NMR spectrometer equipped with a diffusion-specific probehead. The evaluation of the 3D structure of the TM2:TM3 pair in SDS micelle environment was performed based on ^1^H-^1^H NOESY constraints obtained from our previous studies.^16,17^ Heteronuclear ^1^H-^15^N HSQC and ^15^N-edited NOESY data sets were recorded for the TM2 and TM3 peptides containing ^15^N-labeled alanines – Ala80, Ala88 (TM2) and Ala223, Ala231 (TM3). The ^15^N relaxation measurements (*R*_1_, *R*_2_, and ^1^H-^15^N *NOE*) were conducted at 303 K on an 18.8 T magnetic field. All applied procedures are described in detail in the S1 File.

The 25 ns trajectory of molecular dynamic simulations in water bath using TAV protocol was applied to evaluate the 3D structure of TM2:TM3 pair in an SDS micelle. Simulations were performed in Amber 9 software using the param99 force field. Resulted structures were analyzed using the ptraj program included in the Amber software bundle.

**Associated Content**

**Supporting Information**

**S1 File. Detailed description of FRET, NMR and MD results.** Binding parameters for transmembrane TM2 and TM3 segments in SDS micelle evaluated on the basis of FRET experiments (Table A). Hydrodynamic parameters of SDS micelle as well as TM2, TM3 and TM2:TM3 pair in SDS surfactant defined with DOSY spectroscopy at 292 K (Table B). ^15^N relaxation rates for ^15^N-labeled alanines in TM2, TM3 peptides and TM2:TM3 pairs extracted from NMR data acquired in 18.8 T magnetic field (Table C). Comparison of the CD spectra of TM2, TM3 and TM2:TM3 mixture in SDS micellar environment (Figure A, left). The content of α-helical conformation calculated on the basis of CD spectra for the TM2, TM3 and TM2:TM3 under different experimental conditions using a CONTINLL method (Figure A, right). Distance between Pro85 and Pro231 in TM2 and TM3 fragments, respectively, of BTL protein. Results were extracted from 25 ns trajectory of MD dynamic simulations performed with AMBER 9.0 package using parm99 force field (Figure B). Overlay of ^1^H-^15^N HSQC spectra acquired for whole TM2 on natural abundance of ^15^N izotope (red) and TM2 contained ^15^N-labeled alanines (blue) (Figure C, left); overlay of ^1^H-^15^N HSQC spectra acquired for whole TM3 on natural abundance of ^15^N isotope (red) and TM3 contained ^15^N-labeled alanines (blue)(Figure C, right); part of ^1^H-^1^H NOESY spectrum acquired for TM2:TM3 pair. The contact between HA Leu230 and HA Pro231 confirmed existence of cis conformation of Leu230 – Pro231 peptide bond is highlighted (Figure C, bottom). FT PGSE analysis of DPFGDSTE experiment performed for SDS micelle, TM2, TM3 peptides and TM2:TM3 mixture. The extracted value of translational diffusion coefficients is D_tr_ = 5.6 ± 0.4 10^-11^ (m^2^/s) which corresponds to R_h_ ~39 Å for TM2, TM3, and TM2:TM3, respectively (Figure D). Spatial structure of studied species evaluated from NMR data. **A)** SDS micelle; **B)** TM2 trans-membrane fragment of BTL protein in SDS media;^S15^ **C)** TM3 fragment in SDS micelle;^S14^ **D)** TM2:TM3 mixture in SDS environment (Figure E). Experimental *R_1_* and *R_2_* relaxation rates for ^15^N-labeled alanines in TM2 fragment recorded on 18.8 T. Normalized relaxation curves for ^15^N *R_1_* (up) and *R_2_* (down) are demonstrated. The data for TM2 alone and for TM2:TM3 pair are shown in red and blue, respectively (Figure F)*.* Experimental *R_1_* and *R_2_* relaxation rates for ^15^N-labeled alanines (Ala225 and Ala233) in TM3 transmembrane fragment recorded on 18.8 T. Normalized relaxation curves for ^15^N *R_1_* (up) and *R_2_* (down) are demonstrated. The data for TM3 alone and for TM2:TM3 pair are shown in red and blue, respectively (Figure G). Experimental *R_1_* and *R_2_* relaxation rates for ^15^N-labeled alanines (Ala225' and Ala233') in minor conformation of the TM3 transmembrane fragment (cis isomer of Leu230 – Pro231 backbone bond) recorded on 18.8 T. Normalized relaxation curves for ^15^N *R_1_* (up) and *R_2_* (down) are demonstrated. The data obtained for TM3 and TM2:TM3 pair are shown in red and blue, respectively (Figure H). Experimental values of ^15^N R_1_, R_2_ relaxation rates and {^1^H}-^15^N NOE determined on 18.8 T at 303 K for TM2 (black), TM3 (red), and TM2:TM3 (green) pair in SDS-d_25_ micelle. The most right experimental data correspond to the minor conformation of TM3 and TM2:TM3 pair (Figure I). Results of analysis of ^15^N relaxation data for TM2 and TM3 fragments with *Model Free* approach. The *S*^2^, and *R*_1_/R_2_ values determined for TM2 (black), TM3 (red), and TM2:TM3 (green) in SDS-d_25_ micelle are presented. The right most experimental data correspond to the minor conformation of TM3 and TM2:TM3 pair (Figure J).

This material is available free of charge via internet at http://pubs.acs.org.

**Author Information**

***Corresponding Authors**

^*^E-mail: [marjana.novic@ki.si](mailto:marjana.novic@ki.si) (idea, project leader)

^*^ [igor@ibb.waw.pl](mailto:igor@ibb.waw.pl) (for NMR part)

^*^ [spassamonti@units.it](mailto:spassamonti@units.it) (for BTL background studies)

**Notes**

The authors declare no competing financial interest.

**Acknowledgments**

The authors acknowledge the financial support of the Ministry of Higher Education, Science and Technology of the Republic of Slovenia (P1-017 and J1-2151), as well as the support from the European Regional Development Fund (Cross-border Cooperation Programme Italy-Slovenia 2007–2013, TRANS2CARE strategic project). Financial support from the National Centre for Research and Development under research grant “Nanomaterials and their application to biomedicine”, contract number PBS1/A9/13/2012, is gratefully acknowledged (SJ, KS and IZ). The MD simulations of TM2:TM3:SDS complex in water bath were carried out in the Academic Computer Centre (TASK) in Gdańsk, Poland. AS was funded by the R01 GM083960 grant from NIH.

**References**

1. Tzakos AG, Briasoulis E, Thalhammer T, Jäger W, and Apostolopoulos V. (2013) Novel oncology therapeutics: Targeted drug delivery for cancer. J Drug Deliv. 2013. DOI: 10.1155/2013/918304.
2. Buxhofer-Ausch V, Secky L, Wlcek K, Svoboda M, Kounnis V, Briasoulis E, et al. (2013) Tumor-specific expression of organic anion-transporting polypeptides: transporters as novel targets for cancer therapy. J Drug Deliv. 2013. DOI: 10.1155/2013/863539.
3. Baker M. Making membrane proteins for structures: a trillion tiny tweaks. Nature Methods 2010;7: 429 – 434.
4. Passamonti S, Terdoslavich M, Franca R, Vanzo A, Tramer F, Braidot E, et al. Bioavailability of flavonoids: a review of their membrane transport and the function of bilitranslocase in animal and plant organisms. Curr Drug Metab. 2019;10: 369-394.
5. Terdoslavich M, de Graaf IA, Proost JH, Cocolo A, Passamonti S, Groothuis GM. Bilitranslocase is involved in the uptake of bromosulfophthalein in rat and human liver. Drug Metab Lett. 2012;6: 165-173.
6. Passamonti S, Terdoslavich M, Margon A, Cocolo A, Medic N, Micali F, et al. Uptake of bilirubin into HepG2 cells assayed by thermal lens spectroscopy. Function of bilitranslocase. FEBS J. 2005;272: 5522-5535.
7. Montanic S, Terdoslavich M, Rajcevic U, Leo LD, Bonin S, Serbec VC, et al. Development and characterization of a novel mAb against bilitranslocase - A new biomarker of renal carcinoma. Radiology and Oncology 2013;47: 128–137.
8. Župerl Š, Fornasaro S, Novič M, Passamonti S. Experimental determination and prediction of bilitranslocase transport activity. Anal Chim Acta 2011;705: 322-333.
9. Battiston L, Passamonti S, Macagno A, Sottocasa GL. The bilirubin-binding motif of bilitranslocase and its relation to conserved motifs in ancient biliproteins. Biochem Bioph Res Co 1998;247: 687–692.
10. Passamonti S, Battiston L, Sottocasa GL. Bilitranslocase can exist in two metastable forms with different affinities for the substrates. Evidence from cysteine and arginine modification. Eur J Biochem. 1998;253: 84–90.
11. Karawajczyk A, Drgan V, Medic N, Oboh G, Passamonti S, Novic M. Properties of flavonoids influencing the binding to bilitranslocase investigated by neural network modeling. Biochem Pharmacol. 2007;73: 308–320.
12. Passamonti, S., Cocolo, A., Braidot, E., Petrussa, E., Peresson, C., Medic, N., et al. Characterization of electrogenic bromosulfophthalein transport in carnation petal microsomes and its inhibition by antibodies against bilitranslocase. FEBS J. 2005;272: 3282–3296.
13. Maestro A, Terdoslavich M, Vanzo A, Kuku A, Tramer F, Nicolin V, et al. Expression of bilitranslocase in the vascular endothelium and its function as a flavonoid transporter. Cardiovasc Res. 2010;85: 175–183.
14. Ziberna L, Tramer F, Moze S, Vrhovsek U, Mattivi F, Passamonti S. Transport and bioactivity of cyanidin 3-glucoside into the vascular endothelium. Free Radical Bio Med. 2012;52: 1750–1759.
15. Roy Choudhury A, Novič M. Data-driven model for the prediction of protein transmembrane regions. SAR QSAR Environ Res. 2009;20: 741–754.
16. Perdih A, Roy Choudhury A, Zuperl S, Sikorska E, Zhukov I, Solmajer T, et al. Structural analysis of a peptide fragment of transmembrane transporter protein Bilitranslocase. PLoS One 2012;7: e38967.
17. Roy Choudhury A, Perdih A, Zuperl S, Sikorska E, Solmajer T, Jurga S, et al. Structural elucidation of transmembrane transporter protein Bilitranslocase: Conformational analysis of the second transmembrane region TM2 by molecular dynamics and NMR spectroscopy. BBA Biomembrane 2013;1828: 2609–2619.
18. Pieper U, Schlessinger A, Kloppmann E, Chang GA, Chou JJ, Dumont M, et al. Coordinating the impact of structural genomics on the human a-helical transmembrane proteome. Nat Struct Mol Biol 20, 135-138, 2013.
19. Vashisth H, Storaska AJ, Neubig RR, Brooks CL. Conformational Dynamics of a Regulator of G‑Protein Signaling Protein Reveals a Mechanism of Allosteric Inhibition by a Small Molecule. ACS Chem Biol. 2013;8: 2778−2784.
20. Schmidpeter PA, Schmid FX. Molecular determinants of a regulatory prolyl isomerization in the signal adapter protein c-CrkII. ACS Chem Biol. 2014;16: 1145–1152.
21. Carpenter EP, Beis K, Cameron AD, Iwata S. Overcoming the challenges of membrane protein crystallography. Curr Opin Struct Biol. 2008;18: 581–586.
22. Nugent T, Jones DT. Membrane protein structural bioinformatics, J Struct Biol. 2012;179: 327-37.
23. Shen MY, and Sali A. Statistical potential for assessment and prediction of protein structures. Protein Sci. 2006;15: 2507–2524.
24. Russel D, Lasker K, Webb B, Velázquez-Muriel J, Tjioe E, Schneidman-Duhovny Det al. Putting the pieces together: integrative structure determination of macromolecular assemblies. PloS Biol. 2012;10: e1001244.
25. Lo A, Chiu YY, Rødland EA, Lyu PC, Sung TY, Hsu WL. Predicting helix-helix interactions from residue contacts in membrane proteins. Bioinformatics 2009;25: 996–1003.
26. Lia E, Wimleyb WC, Hristova K. Transmembrane helix dimerization: Beyond the search for sequence motifs. BBA Biomembrane 2012; 1818: 183–193.
27. Senesa A, Gersteina M, Engelman DM. Statistical analysis of amino acid patterns in transmembrane helices: the GxxxG motif occurs frequently and in association with β-branched residues at neighboring positions. J Mol Biol. 2000;296: 921–936.
28. Lomize MA, Lomize AL, Pogozheva D, Mosberg HI. OPM: Orientation of Proteins in Membrane database. Bioinformatics 2006;22: 623–625.
29. Gront D, Kmiecik S, Kolinski A. Backbone building from quadrilaterals: A fast and accurate algorithm for protein backbone reconstruction from alpha carbon coordinates. J. Comput. Chem. 2007;28: 1593–1597.
30. Krivov GG, Shapovalov MV, Dunbrack Jr. RL. Improved prediction of protein side-chain conformations with SCWRL4. Proteins 2009;77: 778–795.
31. Jarvet J, Danielson J, Damberg P, Oleszczuk M, Graslund A. Positioning of the Alzheimer Aβ (1-40) peptide in SDS micelles using NMR and paramagnetic probes, J Biomol NMR, 2007;39: 63-72.
32. Kelly SM, Price NC. The use of circular dichroism in the investigation of protein structure and function. Curr Protein Pept Sci. 2000;1: 349–384.
33. Hicks RP, Mones E, Kim H, Koser BW, Nichols DA, Bhattacharjee AK. Comparison of the conformation and electrostatic surface properties of magainin peptides bound to sodium dodecyl sulfate and dodecylphosphocholine micelles. Biopolymers 2003;68: 459–470.
34. Case DA, Darden TA, Cheatham TE, Simmerling CL, Wang J, Duke R, et al. AMBER 9. University of California, San Francisco, 2006.
35. Lipari G, Szabo A. Nuclear magnetic resonance relaxation in nucleic acid fragments: models for internal motion. Biochemistry 1981;20: 6250–6256.
36. Adamian L, Liang J. Helix-helix packing and interfacial pairwise interactions of residues in membrane proteins. J Mol Biol. 2001;311: 891–907.
37. Walters RFS, DeGrado WF. Helix-packing motifs in membrane proteins. Proc Natl Acad Sci. 2006;12: 13658–136563.

***Table 1.*** The distribution of conformations of BTL transmembrane regions among the six different arrangement types.

| Arrangement | |  |  | No. of conformations | |
| --- | --- | --- | --- | --- | --- |
| Rank | Type | | From all 3520 | | From 100 top-scoring |
| 1 | *ABDC (Top2,3,4)^#^* | | 1330 | | 44 |
| 2 | *ACBD (Top5)^#^* | | 862 | | 27 |
| 3 | *ADCB (Top1)^#^* | | 778 | | 15 |
| 4 | *ABCD* | | 281 | | 9 |
| 5 | *ADBC* | | 213 | | 4 |
| 6 | *ACDB* | | 56 | | 1 |
|  |  | | ∑=3520 | | ∑=100 |

*^#^* The five top-scoring (DOPE) conformations of BTL transmembrane regions are shown in Fig. 1B.

**F**i**gure captions**

**Fig. 1.** **A)** The six probable arrangements of the four transmembrane domains of BTL. The transmembrane regions TM1 (*A*), TM2 (*B*), TM3 (*C*) and TM4 (*D*) are represented by the red, green, blue and yellow circles, respectively. The solid lines represent the sequence connectivity, whereas the dotted lines show predicted interactions. **B)** The five top-scoring conformations of BTL transmembrane regions. **C)** The helix-wheel plots of the four transmembrane regions arranged in the ABDC arrangement. The most hydrophobic and hydrophilic residues are colored as green and red, respectively. The charged residues are colored as purple.

**Fig. 2.** Distances between the transmembrane regions TM2 and TM3 for all the conformations in the three most populated arrangement types *ABDC, ACBD* and *ADCB*. **A)** Pro85-Pro231 distances. **B)** Distances between the N-termini of the TM2/3 helices. The best scoring conformation in the most populated arrangement type *ABDC* (Top2) shows **C)** 15.6 Å Pro85-Pro231 distance and **D)** 36.1 Å N-termini distance.

**Fig. 3.** Titration curve for FRET experiments are presented as a ratio between the donor emission at 523 *nm* and the acceptor emission at 627 *nm*.

**Fig. 4.** Ribbon presentation of TM2:TM3 pair in SDS micelle evaluated during 25 *ns* molecular dynamic simulations. The TM2 and TM3 are highlighted as blue and green, respectively; 60 SDS molecules forming the micelle are shown in light grey.

**Fig. 5.** **A)** The 2D ^1^H-^15^N HSQC spectrum of TM2:TM3 pair in SDS-*d*_25_ micelle. The assignment of ^15^N-labeled alanines in TM2 and TM3 fragments are presented together with signals (Ala225' and Ala233') coming from minor populated TM3 conformation; **B)** The 3D structure of TM2:TM3 assembly in SDS surfactant is presented. The position of ^15^N nuclei in ^15^N-labeled alanines incorporated into TM2 and TM3 are depicted as balls. The highly mobile Ala225 is highlighted in blue.
